# Improving accuracy of the intermediate splint in substantial intermaxillary sagittal discrepancies using an extra anterior anchorage point: technical note

**DOI:** 10.4317/medoral.24089

**Published:** 2020-08-27

**Authors:** Federico Hernández-Alfaro, Victoria Rosemberg, Jorge Masià-Gridilla, Adaia Valls-Ontañón

**Affiliations:** 1 MD, DDS, PhD, FEBOMS. Chief, Maxillofacial Institute, Teknon Medical Center Barcelona, Barcelona, Spain; 2 MD, DDS, PhD, FEBOMS. Professor and Department Head, Department of Oral and Maxillofacial Surgery, Universitat Internacional de Catalunya, Sant Cugat del Vallès, Barcelona, Spain; 3 MD. MD Fellow, Institute of Maxillofacial Surgery, Teknon Medical Center Barcelona, Barcelona, Spain; 4 MD, FEBOMS. Attending surgeon, Maxillofacial Institute, Teknon Medical Center Barcelona, Barcelona, Spain; 5 MD, FEBOMS. Collaborator Teacher, Department of Oral and Maxillofacial Surgery, Universitat Internacional de Catalunya, Sant Cugat del Vallès, Barcelona, Spain; 6 MD, DDS, PhD, FEBOMS. Associate Professor, Department of Oral and Maxillofacial Surgery, Universitat Internacional de Catalunya, Sant Cugat del Vallès, Barcelona, Spain

## Abstract

**Background:**

To describe a technical feature that increases the stability of the intermediate splint in patients where bimaxillary surgery with great maxillary/mandibular advancements are planned.

**Material and Methods:**

Prospective evaluation of the intermediate splint dental vertical penetration in patients undergoing bimaxillary surgery where great sagittal discrepancy occur in the anterior sector between the upper and lower jaws when the intermediate splint is placed by adding an extra intermaxillary fixation (IMF) screw (2x9 mm) placed between the central incisors of the maxilla and fixed to the most anterior aspect of the intermediate splint following the direction of the sagittal maxillo-mandibular discrepancy from January to September 2018.

**Results:**

The postoperative evaluation comparing the accuracy of conventional fixation versus fixation with an extra anterior anchorage point through photographic assessment and intraoral digital scanner demonstrated better dental penetration, and therefore improved intermediate splint precision with the latter in all cases

**Conclusions:**

Our results suggest that this is a simple and safe technique that can be easily reproduced and optimizes the outcomes by increasing the accuracy of translation of the planned surgical movements to the operating room.

** Key words:**Orthognathic surgery, intermediate splint, accuracy, intermaxillary fixation, bone screw.

## Introduction

Since the introduction of rigid internal fixation methods, successful intraoperative stability in orthognathic surgery mainly depends on both surgical planning and the subsequent surgical technique employed (1). Consequently, it is essential to accurately transfer such planning to the surgical procedure. The current gold standard is to translate surgical planning through surgical splints (2-4). In bimaxillary surgeries, an intermediate splint is used first to guide the movement of one maxilla relative to the other that has not yet been mobilized. Then, a final splint guides the movement of the other jaw and secures final occlusion. Regardless of which bone is repositioned first, precise repositioning is imperative, because once stabilized, it becomes the reference for repositioning the other bone. Hence, precise designing of the splint is as important as its correct and sTable adaptation in the intraoperative period (5).

In patients where bimaxillary surgery with great maxillary/mandibular advancements are planned, sagittal discrepancy may occur in the anterior sector between the upper and lower jaws when the intermediate splint is placed. Conventional anterior wiring through the hooks of dental arches in these situations often causes imbalance and inadequate vertical penetration of the teeth into the splint and may pull the braces off the teeth. Since conventional anterior wiring does not provide enough vertical stability and penetration of the teeth into the splint of the teeth.

Such instability could lead to a wrong bone position prior to internal rigid fixation with miniplates and screws, and consequently to defective positioning of the complementary maxillary bone (6).

The present article describes a technical feature that increases the stability of the intermediate splint in cases where substantial intermediate sagittal discrepancy could lead to unreliable positioning of the first mobilized maxillary bone, thereby guiding osteosynthesis more precisely.

## Material and Methods

- Patient selection 

This technique was implemented in patients undergoing bimaxillary surgery where great sagittal discrepancy occurred in the anterior sector between the upper and lower jaws when the intermediate splint was placed - with subsequent inadequate vertical penetration of the teeth into the splint despite conventional intermaxillary fixation, which led to surgical instability. The patient recruitment period was from January to September 2018.

The Ethics Commitee at Teknon Medical Center approved the study under number ISF. The Declaration of Helsinki guidelines were followed in all treatment phases, and written informed consent was obtained from all subjects and from all patient images submitted.

- Virtual planning work-up

**Figure 1:**
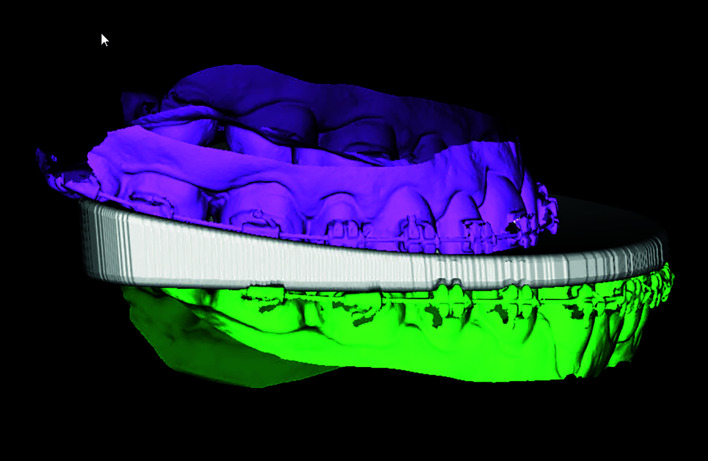
Intermaxillary sagittal discrepancy and intermediate splint are shown in virtual orthognathic surgery planning.

Repositioning of the maxillo-mandibular complex was planned following the upper incisor to soft tissue plane (UI-STP) protocol, previously validated and described in detail elsewhere (7). Then, surgery was virtually planned using a specific software (6,8) (Dolphin® 3D Orthognathic Surgery Planning Software Version 11.8, Pentium 4 Processor 3.8 GZ, W/SP5 Windows © XP Professional, 120 GB memory, 2 GB RAM), and production of the splints was carried out with a 3D printer (TierTime UP Box3D) (Fig. 1).

- Surgical technique

**Figure 2:**
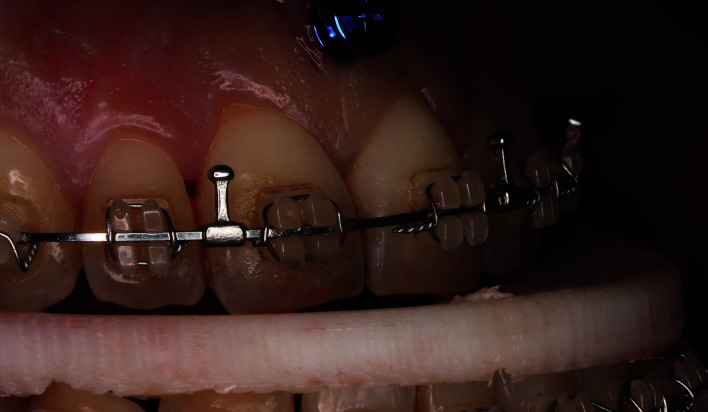
Lack of complete penetration of the anterior teeth into the intermediate splint and vertical balancing movement after conventional fixation.

All patients were operated upon under general anesthesia by the same surgeon (FHA) according to the mandible first protocol. Once bilateral sagittal split osteotomy was performed, the intermediate splint was placed between the dental arches, and intermaxillary fixation was achieved with 0.12 mm wires. In all cases the intermediate splint presented vertical balancing movement and anteroposterior instability, making it impossible to achieve complete penetration of the anterior teeth into the splint (Fig. 2).

**Figure 3:**
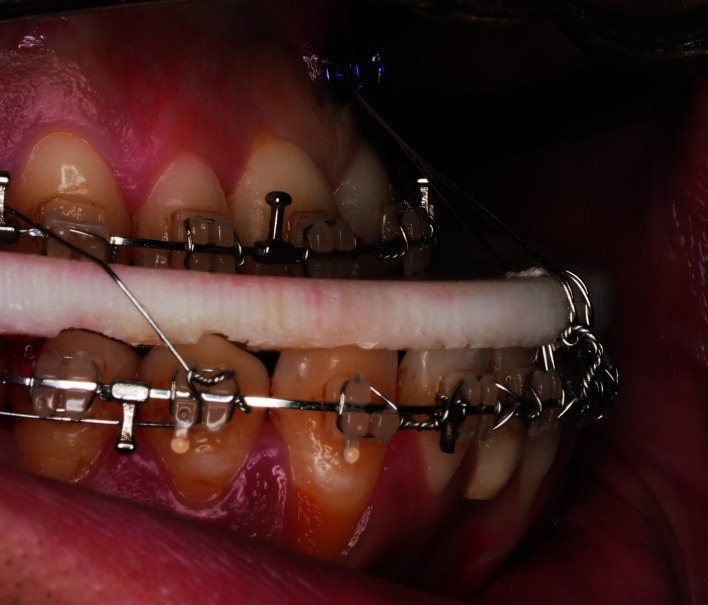
Fixation of the intermediate splint to the upper screw and to the lower arch with two extra wires improves intermediate splint accuracy.

In order to allow full stability and dental penetration into the intermediate splint, an intermaxillary fixation (IMF) screw (2x9 mm) was placed in the maxilla between the central incisors at the upper level of the mucogingival junction, avoiding damage to the roots of the neighboring teeth. The authors recommend not introducing the screw completely, so that the head of the screw is at the same level as the edge of the surgical splint in order to assure a vertical traction and a complete penetration of the teeth into the splint. Then, a tunnel was made in the central area of the most anterior aspect of the intermediate splint using a fissure burr, following the direction of the sagittal maxillo-mandibular discrepancy, and then the intermediate splint was fixed to the screw and to the lower arch with two extra wires (Fig. 3).

Application of this technical feature improved teeth penetration into the splint and solved the abovementioned intermediate splint balancing in all cases, ensuring its complete stabilization in the sagittal plane.

Then, rigid internal fixation with a hybrid technique (one miniplate fixed with 4 monocortical screws and a retromolar bicortical screw) was carried out, followed by IMF removal (9). Before removing the intermediate splint, proper condylar positioning and intermediate occlusion were checked, and finally both the intermediate splint and the midline screw were removed. Lastly, the upper maxilla was repositioned according to the final splint.

- Evaluation

In order to objectively evaluate the stability and accuracy of the intermediate splint, intraoral pictures were obtained intraoperatively at two specific time points: after conventional intermediate splint fixation and after placing the extra anterior anchorage point in all cases (Fig. 2, Fig. 3).

**Figure 4:**
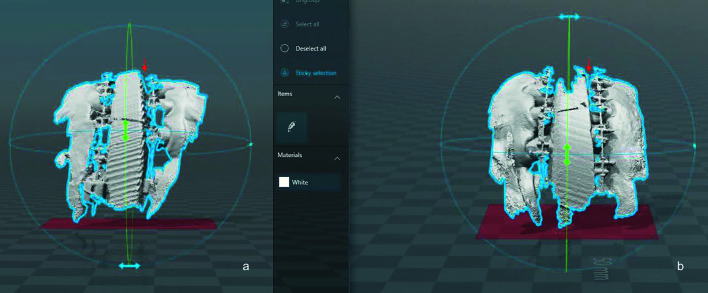
Comparison of both intraoperative digital scans (a) after conventional intermediate splint fixation; (b) after placing the extra anterior anchorage point.

In addition to the photographic assessment, a dental arch anatomy registry was obtained intraoperatively using digital scanning (Lava Scan ST Scanner; 3M ESPE, Ann Arbor, MI, USA) at the same two specific time points in four patients who had undergone surgery during the last period of the study (between July and September 2018). Thus, better dental penetration into the intermediate splint could be proven when comparing both full digital records (Fig. 4).

## Results

The technique was applied in 11 of the 88 patients scheduled for orthognathic surgery for the correction of a dentofacial deformity between January and September 2018. The studied sample comprised 7 women and 4 men with a mean age of 26 years (range 18-39). All patients presented a maxillo-mandibular biretrusive profile; 5 of them with class II and 6 with class III according to Angle’s occlusion classification (Table 1).

The sagittal gap between the upper and lower central incisors when the intermediate splint was placed intraoperatively was measured, obtaining a gap of 11 mm (range 8-13 mm) (Table 1). The mean increase in surgery time was three minutes, surgeon confidence in intermediate splint stability was excellent, and no complications such as root damage or hardware breakage were reported (Table 1).

The postoperative evaluation comparing the accuracy of conventional fixation versus fixation with an extra anterior anchorage point through photographic assessment and intraoral digital scanner demonstrated better dental penetration, and therefore improved intermediate splint precision with the latter in all cases (Table 1).

## Discussion

Because of the lack of full accuracy of intermaxillary splints in orthognathic surgery, several alternatives have been proposed such as intraoperative guidance via a real-time navigation system (10) or custom-machined miniplates with bone-supported guides (11). Nevertheless, no method has been shown to surpass the splint-encoded gold standard in terms of precision.

Moreover, in terms of translation of treatment plan precision, a previous article by our group already showed 3D planning and CAD/CAM generation of surgical splits to be accurate to tenths of a millimeter (6,8). However, this is based on the assumption that intraoperatively all teeth are perfectly inserted in the splint, which is especially difficult to achieve in cases where the mandible first protocol is applied and the anterior segment of this bone is repositioned far away from the upper maxilla. In fact, this article reported the “z” (vertical) axis to be the least accurate, precisely due to incomplete dental insertion into the splint (8).

Regarding the increase in surgery time, it could have been longer without this technical detail, due to intraoperative instability and a lack of overall accuracy – thus implying the eventual need to redo osteosynthesis or even reoperate the patient in a second stage.

Although we advocate the mandible first protocol, this technique can also be helpful when using the maxilla first protocol, in cases where substantial anterior sagittal discrepancy is expected when placing the intermediate splint, such as in biretrusive class II patients. After Le Fort I osteotomy, the intermediate splint would be placed in position to guide maxillary advancement, and intermaxillary wiring would be performed afterwards. In order to increase the stability of the intermediate splint, the IMF screw would be placed on the midline of the mandible in order to fix it to the intermediate splint with two extra wires, as previously described.

It thus may be concluded that this technique can be indicated in all cases where placing the intermediate splint is associated with significant anterior maxillo-mandibular discrepancy in the sagittal plane. In such cases, the described technique seeks to enhance the stability and consequently the reliability of the intermediate splint, allowing correct repositioning and fixation of the first mobilized jaw and subsequent suiTable mobilization of the other jaw.

In sum, this is a simple and safe technique that can be easily reproduced by other surgeons and which optimizes the outcomes by increasing the accuracy of translation of the surgical virtually or conventionally planned movements to the operating room. We underscore the importance of paying special attention to not damage the roots of the neighboring teeth when placing the IMF screw.

**Table 1:**
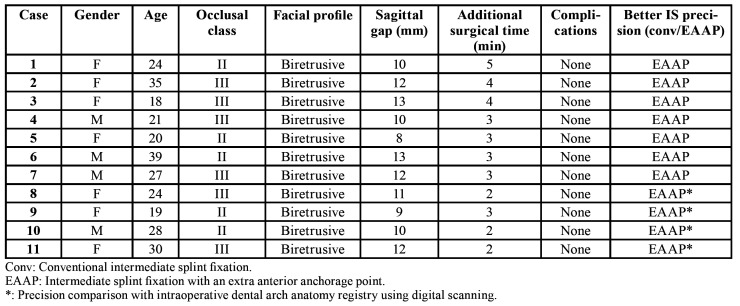
Descriptive analysis and results of the study sample.
